# A New Tool for Nutrition App Quality Evaluation (AQEL): Development, Validation, and Reliability Testing

**DOI:** 10.2196/mhealth.7441

**Published:** 2017-10-27

**Authors:** Kristen Nicole DiFilippo, Wenhao Huang, Karen M Chapman-Novakofski

**Affiliations:** ^1^ Division of Nutritional Sciences University of Illinois at Urbana-Champaign Urbana, IL United States; ^2^ Department of Education Policy, Organization and Leadership University of Illinois at Urbana-Champaign Champaign, IL United States; ^3^ Department of Food Science and Human Nutrition Division of Nutritional Sciences University of Illinois at Urbana-Champaign Urbana, IL United States

**Keywords:** evaluation, mobile apps, dietitians, health education, diet, food, and nutrition

## Abstract

**Background:**

The extensive availability and increasing use of mobile apps for nutrition-based health interventions makes evaluation of the quality of these apps crucial for integration of apps into nutritional counseling.

**Objective:**

The goal of this research was the development, validation, and reliability testing of the app quality evaluation (AQEL) tool, an instrument for evaluating apps’ educational quality and technical functionality.

**Methods:**

Items for evaluating app quality were adapted from website evaluations, with additional items added to evaluate the specific characteristics of apps, resulting in 79 initial items. Expert panels of nutrition and technology professionals and app users reviewed items for face and content validation. After recommended revisions, nutrition experts completed a second AQEL review to ensure clarity. On the basis of 150 sets of responses using the revised AQEL, principal component analysis was completed, reducing AQEL into 5 factors that underwent reliability testing, including internal consistency, split-half reliability, test-retest reliability, and interrater reliability (IRR). Two additional modifiable constructs for evaluating apps based on the age and needs of the target audience as selected by the evaluator were also tested for construct reliability. IRR testing using intraclass correlations (ICC) with all 7 constructs was conducted, with 15 dietitians evaluating one app.

**Results:**

Development and validation resulted in the 51-item AQEL. These were reduced to 25 items in 5 factors after principal component analysis, plus 9 modifiable items in two constructs that were not included in principal component analysis. Internal consistency and split-half reliability of the following constructs derived from principal components analysis was good (Cronbach alpha >.80, Spearman-Brown coefficient >.80): behavior change potential, support of knowledge acquisition, app function, and skill development. App purpose split half-reliability was .65. Test-retest reliability showed no significant change over time (*P*>.05) for all but skill development (*P*=.001). Construct reliability was good for items assessing age appropriateness of apps for children, teens, and a general audience. In addition, construct reliability was acceptable for assessing app appropriateness for various target audiences (Cronbach alpha >.70). For the 5 main factors, ICC (1,k) was >.80, with a *P* value of <.05. When 15 nutrition professionals evaluated one app, ICC (2,15) was .98, with a *P* value of <.001 for all 7 constructs when the modifiable items were specified for adults seeking weight loss support.

**Conclusions:**

Our preliminary effort shows that AQEL is a valid, reliable instrument for evaluating nutrition apps’ qualities for clinical interventions by nutrition clinicians, educators, and researchers. Further efforts in validating AQEL in various contexts are needed.

## Introduction

Smartphone ownership reached 68% of Americans in 2015, increasing from 35% in 2011 [1]. Smartphones allow instant access to health information, enabling 62% of smartphone owners who obtain information on health conditions via smartphone [2]. A nationwide survey corroborated these results, showing 58% of mobile phone users in the United States had downloaded a health app, citing tracking physical activity (52.8%), tracking diet (46.6%), weight loss (46.8%), and to learn exercises (34.0%) as the most common reasons for health app use [3]. The study further suggested that research is needed to create methods to evaluate health app quality to ensure the needs of app users are met [3].

Dietitians are using apps in practice; a 2012 survey of Canadian dietitians showed 57.3% of dietitians surveyed used apps in practice, and 83.6% of those not currently using apps expressed interest in future app use in dietetic practice [4]. Whereas nutrition-related health apps are widely available and utilized, health professional’s involvement in the development of apps’ content and functionalities remains uncertain [5]. Currently, no method grounded in empirical studies for evaluating and selecting apps specifically for use in nutrition interventions exists. When selecting an app for dietetic practice, dietitians resort to subjectively relying on best clinical judgment or relying on similarly subjective recommendations of others [6].

Standardized app evaluation is called for to present cost-effective, transparent means of providing app developers and distributors with the necessary information to guide app selection [7]. A need for a systematic framework for evaluating health-based apps [8] and weight loss apps [9] have both been emphasized, although a recent investigation into best practices in health app evaluations emphasizes that an available best practice approach could not be identified [10]. The study did identify various constructs evaluated in studies, suggesting that a review of apps should include an evaluation of usability or functionality, a critique of potential to promote behavior change, and the quality of the health-related content [10]. None of the reviewed studies included an evaluation of all three constructs [10]. Whereas studies evaluating nutrition apps have reported the use of evidence-based treatment strategies [11], the use of theory [12], as well as behavior change techniques within apps [11,13-15], a measure of quality as perceived by the health care provider is needed, which evaluates the quality of the content and the functionality of the app to complement previous work evaluating scientific evidence that goes into mobile app development. 

With this in mind, the objective of this study was the development, validation, and reliability testing of the app quality evaluation (AQEL) tool, an evaluation instrument for judging the quality of apps to aid in the development and selection of apps for nutrition interventions.

## Methods

### Survey Development

PubMed was searched first for nutrition and health education apps evaluation studies and then for educational website evaluation studies to form a pool of initial survey items. Search terms included website, app, and evaluation. The search was not limited by date. No apps evaluation studies were found, but 6 studies were identified that evaluated websites [13,16-20]. Three studies were excluded after review by 2 researchers. Two of these assessed only for the inclusion of behavior theories rather than broader measures of quality [13,19] and another did not provide specific questions within the paper [20]. The 3 evaluation tools selected to create the initial item pool were chosen based on relevance to education and coverage of items targeting the areas of content, usability, and technology [16-18].

Ninety-four items from these 3 selected website evaluation tools, with n=43 in one [16], n=16 in another [17], and n=35 in the third [18] were entered into a spreadsheet and sorted based on relevance to three categories: content, usability, and technology. One researcher completed the initial sorting of questions, with a second researcher reviewing the category selections. Any disagreement was discussed until agreement was reached. These categories were selected to broadly cover the needs of previously identified stakeholders in nutrition app development and use, namely the researchers and practitioners involved in content selection and distribution of the app as an educational tool [6], the end users (potential patients or clients) of the app in terms of usability [6,7], and developers of the app technology [7]. The 2 researchers removed items specific to websites with no relevance to apps, reworded other items to pertain to apps and nutrition, and divided complex questions into 2 or more questions. This resulted in 27 content, 9 technology, and 19 usability items. Additional items were created based on specific features of apps, including transition between pages and touchscreen functionality [21], with 6 new content items, 8 new technology items, 8 new usability items, and 2 questions identifying the app and the device used to download the app. As the three sources used different rating scales, questions were converted to 5-point Likert-type scales (content n=26, usability n=19, and technology n=13); yes or no, or yes, maybe, or no (content n=5, usability n=3, and technology n=1); or open ended questions (content n=2, usability n=5, and technology n=1) [15,22].

### Content and Face Validation

Nutrition experts and app developers completed content validation by reviewing survey sections; app end users completed face validation. Institutional review board approval was obtained at all points where participants were involved. A total of 13 nutrition experts, including registered dietitians and nutrition professors with publications in app-based nutrition interventions, were contacted to review the 33 nutrition content questions, with 6 agreeing. Of 15 technology experts contacted, 4 agreed. For face validation, app users were recruited through a Web-based weekly email newsletter at the University. This newsletter targets all university employees, not just academic faculty. The first 14 respondents were requested to review the 27 usability questions; 10 completed the review.

Each expert and app user was asked to review the survey selecting from the following options: complete the survey considering an app used in the past, complete the survey reviewing a new app, or provide general opinions of the survey questions. To specifically improve the validity of the survey, experts and app users were asked to cross out inappropriate questions, circle unclear words or phrases, modify unclear questions, add additional questions they felt would improve the survey, and provide any additional comments on survey items they felt would benefit survey development.

After modification based on expert panel review suggestions, further content and face validation was completed because of the magnitude of the changes and to allow review of the whole tool. Four of six nutrition expert panel reviewers repeated the procedures described above, reviewing all of 51 preliminary AQEL items.

### Item Reduction

To reduce and evaluate the reliability of the preliminary AQEL items, nutrition professionals were recruited via an online discussion group from the Nutrition Education for the Public Dietetics Practice Group of the Academy of Nutrition and Dietetics. A total of 25 nutrition professionals evaluated 3 apps each using the 51 AQEL items. These apps were randomly assigned from a pool of 15 apps selected to represent a wide variety of nutrition-related apps, as described later on. This provided 75 evaluations using the preliminary AQEL items. The nutrition professionals completed a second evaluation of each app 3 weeks later, providing a total of 150 evaluations using the 51 preliminary AQEL items.

App selection specifically targeted 3 categories: popular apps, unpopular apps, and app-based games. Popular and unpopular apps were determined by searching the Apple App Store using 6 terms: healthy eating, nutrition, diet, nutrition games, diabetes, and diabetes recipes. This was completed daily (May 2014 to July 2014; January 2015 to February, 2015). In the App Store, the default setting was changed so that the apps were searched *by popularity*, and daily top apps were recorded. Nine apps ranked in the top 3 for their search term in both the 2014 and 2015 searches. Five of these were selected for reliability testing, including a calorie counter, a nutrition quiz, a digestive system game for kids, and 2 diabetes apps. An additional 4 apps were selected that were considered unpopular. Three had fallen in popularity, ranking in the top 6 of the 2014 search but not appearing in the 2015 search. These were a weight loss hypnosis app, a weight loss app, and a calorie tracker. One additional app was considered unpopular as it was the last English language app listed in a search of nutrition games on June 11, 2015. Six additional apps were selected to increase the number of educational gaming apps because of the specific interest of the research team to better understand educational games.

Item number was reduced first by removing 7 questions where not applicable was selected more than 50% of the time for the 150 evaluations, as the frequent selection of not applicable for a given item indicated that the question was considered by participants as irrelevant for evaluating apps in general. These included questions such as “how well does the app provide capacity to log food?” An additional 10 items were not included in principal component analyses (PCA) that allowed AQEL to be modified for the target audience of the app. Five of these items related to the specific age group the evaluator felt the app targeted, the other 5 to the specific educational needs of the app end user. For these items, the app evaluator selected the groups they would like to evaluate the app for; therefore, limiting the responses to these items.

The 34 remaining items were reduced into categories using PCA with varimax rotation with the 150 app evaluations. Items were removed and analysis rerun when communalities were less than .50. Factor criteria were Eigen values of 1 or more, at least two items per factor, primary loadings of .45 or more [23], and secondary loadings with a difference of at least .20. Additionally, only the number of factors required to explain just over 70% of the variance were retained. Scree plots were also examined for points of inflection to determine which factors to retain. For further refinement of factors, items not meeting these criteria were eliminated and additional factor analyses were run on the remaining factors with the factor number limited to the number of factors identified in the previous analysis.

Multiple imputation with 100 imputations followed by aggregation of imputations was used to treat missing data, with new imputations run each time items were removed.

### Reliability Analysis

Construct reliability of the final factors was assessed using Cronbach alpha. Spearman-Brown coefficient was used to test split-half reliability. For construct reliability only, items not on a 5-point scale were adjusted to a 5-point scale. These analyses were conducted using the first occasion apps were evaluated. Each rater’s first evaluation was used for analysis (n=75).

Items within each factor were summed to create factor scores. Test-retest analysis was conducted comparing first and second evaluations using Wilcoxon sign-rank as the data were not normally distributed (n=75).

Interrater reliability (IRR) for the evaluation of each app using factors identified in PCA was tested using one-way random, average measures intra correlations (ICC) using the first evaluation (n=75).

For the items assessing app appropriateness for various age groups (n=5) and target audiences (n=5), construct reliability was measured using Cronbach alpha. For the questions regarding age group, the second evaluation completed by each evaluator was utilized because of a mistake in the questionnaire discovered after many of the first evaluations had been completed. For the target audience, the first evaluation of the app by each evaluator was used. Sample size varied, as evaluators were able to select the age groups and target audiences. All sample sizes are reported.

Further IRR testing of app evaluations using the factors identified in PCA plus the age and audience constructs utilized two-way random, average measures ICC. For this analysis, a new dataset was collected, with 15 nutrition professionals using the AQEL tool to evaluate MyFitnessPal, the most popular app according to a dietitians’ survey (unpublished data, 2017) [24]. For this analysis, the age group apps evaluated for was *adults* and the evaluators considered the target audience of *people seeking weight loss support* (n=15).

Reporting on the survey using the Checklist for Reporting of Internet E-Surveys can be found in Multimedia Appendix 1 [25]. All statistical analysis were conducted using the Statistical Package for the Social Sciences (SPSS) for Windows, version 24 (IBM Corp).

## Results

### Content and Face Validation

Specific recommendations from the nutrition experts included clarifying words such as aim and target population, changing the rating scales used, and requesting additional items on skill-building and goals of the apps. App users recommended reducing repetitive questions. Technology experts recommended clarification of 10 items and dividing 3 items into multiple questions plus additional items on data storage and user characteristics. The recommendations of the expert panels led to the modification of nearly every item. Once modifications were completed as described, the three subtools were combined into the full AQEL with 51-scaled items for evaluating app quality plus items for app identification. The second expert panel resulted in minor clarifications.

### Item Reduction

For the first round of PCA, Kaiser-Meyer-Olkin measure of sampling adequacy was .59 and the Bartlett test of sphericity was significant (χ^2^_561_=4456, *P*<.001). Correlations were greater than .30, and communalities were greater than .50 for all items. Nine factors had Eigen values greater than 1, but 8 factors explained 73% of the variance. When using 9 factors, 3 items were removed because they loaded onto 2 factors with less than a .20 difference; 1 item was removed because it did not load at .45 on any factor. Removing these items resulted in the elimination of a factor; therefore, the next analysis was run with 8 factors.

**Figure 1:**
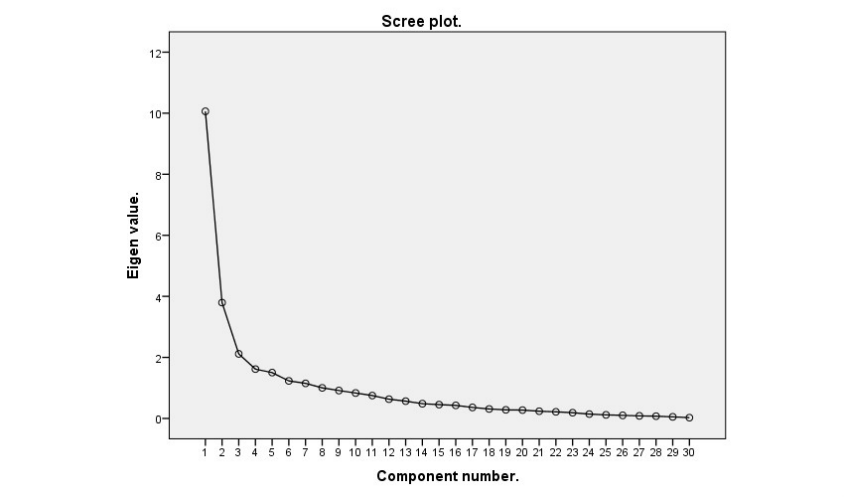
Scree plot for second round of principal components analysis of items assessing nutrition app quality.

In the second PCA analysis, Kaiser-Meyer Olkin measure of sampling adequacy was .67, Bartlett test of sphericity was significant (χ^2^_435_=3357, *P*<.001). The point of inflection on the scree plot indicated that 5 factors should be retained (Figure 1); therefore, analysis was rerun with 5 factors. Five items had communalities below .50; these item were removed, and PCA was completed a third time with 5 factors.

In the final PCA analysis, Kaiser-Meyer-Olkin measure of sampling adequacy was .81, and Bartlett test of sphericity was significant (χ^2^_300_=2929, *P*<.001). All correlations were greater than .30, and communalities were .50 or greater when rounded to the nearest tenth. For items that loaded on more than one factor, differences were greater than .20 when rounded to the nearest tenth, and these items were placed on the factor where they loaded the highest. The final factor loadings are presented in Table 1. 

**Table 1:** Primary factor loadings of items assessing nutrition app quality.

Item		Factor loading value
**Factor 1-Behavior change potential **		
	In your opinion does the app try to change behavior?	.56
	Do you think the app will lead to behavior change?	.81
	When considering activities within the app, will the activities help the user to change behavior?	.80
	Would your friends use this app?	.57
	Do you intend to use this app in the future?	.59
	Will you do something differently after using this app?	.82
	Will you try to do something new after using this app?	.82
**Factor 2-Support of knowledge acquisition **		
	In your opinion, does the app try to increase knowledge?	.78
	Do you think the app will increase the user’s knowledge?	.69
	When considering activities within the app, will the activities help the user to increase knowledge?	.70
	How well does the app provide information?	.71
	How well does the app provide feedback on progress?	.75
	How well does the app provide timely feedback whenever needed?	.77
	Is feedback provided when the user participates in an activity in the app?	.67
**Factor 3-App function **		
	Please rate the speed of loading the app	.62
	Please rate the user’s ability to retrace their steps if they need to	.80
	Please rate the transitions from page to page	.87
	Please rate the function of any animations (quick and functional—slow and fragmented)	.60
	Please rate the design of menus and icons	.79
	Please rate the ease of navigation to the app’s various features	.81
**Factor 4-Skill development **		
	In your opinion does the app try to develop a skill?	.89
	Do you think the app will lead to the development of a skill?	.71
	When considering activities within the app, will the activities help the user to develop a skill?	.70
**Factor 5-App purpose **		
	Do you feel that the app has a clear purpose?	.68
	Does the app title accurately describe the content of the app?	.76

Missing data represented 6.40% (240/3750) of the entries in the dataset. Little missing completely at random (MCAR) test indicated data were not MCAR (χ^2^_1140_=1267, *P*=.005); therefore, multiple imputations with 100 imputations were used to treat missing data.

### Reliability Analysis

Measures of reliability are reported in Table 2. Construct reliability for factors 1 to 4 were all excellent with Cronbach alpha between .80 and .90 [26]. Split-half reliability for factors 1 to 4 were also good with Spearman-Brown coefficients between .80 and .90. For factor 5, Cronbach alpha was not used as there were only two items, and split half reliability was .65.

Test-retest reliability was not significant, indicating that evaluations of each factor did not change over time, with the exception of the factor evaluating the potential of the app assist skill development (Table 3).

IRR for each app was excellent [27]. These results are shown in Table 4. The 15th app was not included, as only one evaluator completed the evaluation for this app.

For the 5 items assessing specific age groups, construct reliability was good except for evaluations specific to adults (Table 5) [26].

For the 5 items assessing the app’s appropriateness of various audience, construct reliability was less than desirable; however, removing one item improved the reliability to be at minimum acceptable (>.70) and for many audiences good (>.80) or excellent (>.90) [19,26]. These results are presented in Table 6.

In the second dataset with 15 nutrition professionals evaluating My Fitness Pal, two-way random ICC using average measures was excellent, with ICC (2,15)=.99, *P*<.001. Single measure ICC was also good, with ICC (2,15)=.83, *P*<.001, reflecting that the AQEL tool, including both the factors identified by PCA and the two additional modifiable constructs, can be reliably used both by averaging responses of multiple evaluators or by a single evaluator.

**Table 2:** Construct and split-half reliability of factors evaluating app quality (n=75 evaluations).

Factor	Construct reliability (Cronbach alpha)	Split-half reliability (Spearman-Brown coefficient)
Factor 1-Behavior change potential	.89	.82
Factor 2-Knowledge	.88	.84
Factor 3-App function	.89	.83
Factor 4-Skill development	.81	.83
Factor 5-App purpose	N/A^a^	.65

^a^Cronbach alpha not applicable as the factor only includes 2 items.

**Table 3:** Test-retest reliability of factors of the app quality evaluation (n=75 evaluations).

Factor or item		Test-retest reliability Wilcoxon signed-rank test (*P* value)
Factor 1-Behavior change potential		.13
Factor 2-Knowledge		.05
Factor 3-App function		.55
**Factor 4-Skill development^a^**		.001
	Skill item 1	.01
	Skill item 2	.006
	Skill item 3	.05
Factor 5-App purpose		.89

^a^Results for individual items shown for skill development as differences were found to be significant.

**Table 4:** Interrater reliability of dietitians evaluating apps using the app quality evaluation (AQEL) tool (n=75 evaluations).

App	ICC^a^(1,k)	*P* value
Calorie Counter by MyFitnessPal	ICC (1,3)=.88	.003
Nutrition Quiz 600+ Facts, Myths and Diet Tips	ICC (1,3)=.94	<.001
Science Heroes: Digestive System for Kids by Yogome Inc.	ICC (1,5)=.86	.001
Diabetes In Check: Coach, Blood Glucose & Carb Tracer by Everyday Health Inc.	ICC (1,8)=.96	<.001
Diabetes App Lite by BHI Technologies, Inc.	ICC (1,6)=.96	<.001
Weight Loss Hypnosis-Free by Surf City Apps LLC	ICC 1,4)=.80	.01
Jillian Michael’s Slim Down	ICC (1,4)=.87	.002
MyPlate Calorie Tracker	ICC 1,5)=.96	<.001
National Center on Health Nutrition Education Gamelettes by ZebraZapps Engineering	ICC (1,5)=.97	<.001
Nutrition and Healthy Eating by Tribal Nova	ICC (1,7)=.95	<.001
Awesome EatsTM by whole Kids Foundation	ICC (1,6)=.95	<.001
Eat Smart by Edin	ICC (1,7)=.92	<.001
Eat & Move O-Matic by Learning Games Lab, NM State University	ICC (1,6)=.96	<.001
Harry’s Healthy Garden	ICC (1,5)=.98	<.001

^a^ICC: intraclass correlations.

**Table 5:** Construct reliability of items assessing app appropriateness for evaluator-selected age groups.

Age group	Evaluations completed (n)^a^	Construct reliability (Cronbach alpha)
Children	36	.82
Teens	12	.86
Adults	31	.53
General audience	10	.80
Other audience	6	N/A^b^

^a^Analysis of responses from evaluators second evaluation of each app because of survey error discovered during first round of evaluations.

^b^Analysis not completed because of negative covariance among items.

**Table 6:** Construct reliability of items assessing app appropriateness for evaluator-selected audiences.

Audience	Evaluations completed	Construct reliability	Construct reliability with item 5 deleted^a^
	(n)	(Cronbach alpha)	(Cronbach alpha)
People seeking help for medical conditions	16	.62	.82
People with specific nutrition concerns	5	.67	.94
People who are shopping for food	3	.40	.98
People seeking recipe or meal ideas	8	.20	.70
People seeking guidance for restaurant eating^b^	1	-	-
People seeking weight loss support	18	.53	.92
People seeking nutrition education	43	.57	.71
Other audience	16	.59	.72

^a^Item 5: Does the level of detail exceed the target populations’ abilities?

^b^Analysis not run as only 1 person selected this option.

## Discussion

### Principal Findings

In summary, the 94 items first selected from the literature were modified to 51 items after expert panel review. Five items evaluating app appropriateness for various age groups and 5 items evaluating app appropriateness for evaluator chosen target audiences were not included in PCA, as the response number to these items was limited. Construct reliability testing of these two constructs resulted in removal of one item evaluating appropriateness for target audiences. This left 41 items to be grouped into factors for evaluating apps. Seven of these were eliminated as raters selected the option of does not apply in more than 50% of the evaluations. Therefore, 34 items were tested using PCA. After three rounds of PCA, the result was a survey with 25 items grouped into 5 factors for evaluating apps, plus 5 additional items that can be used for evaluating app appropriateness for various age groups, and 4 additional items which can be used to evaluate apps for specific target audiences (Multimedia Appendix 2).

The AQEL is a valid, reliable tool for evaluating app quality. Careful consideration of stakeholder needs, including nutrition educators and researchers, app end users, and developers, guided development and assurance of face and content validity. Construct, split-half, test-retest, and IRR were also evaluated to establish the overall reliability of this new tool for use in evaluating nutrition apps.

The validation and reliability testing of AQEL contributes to the literature by providing a standardized method of evaluating and reporting on nutrition apps, a gap identified previously in app research [7-10].

### Limitations

AQEL allows for the evaluator to specifically choose both an age group and audience for some of the evaluation items. Although a strength of the tool, it did limit the samples size for reliability testing of these items. Addressing characteristics of the intended app user, such as learning preferences and skill with technology, is an important aspect of selecting apps. Generally apps are able to accommodate a wide variety of user preferences as they are able to deliver multimedia content based on users’ choices. Rarely do apps only deliver text or multimedia content. Assessment based on age group begins to address variations in app users; however, clinician assessment remains an important piece when selecting apps for clients to account for individual preferences and needs.

App users for face validation were recruited from a population of university employees reflecting a wide range educational experience and income; however, demographic data were not collected from this group.

Rater knowledge of apps is important for completing accurate evaluations. For this reason, raters were asked to spend 10 to 15 min becoming familiar with the app before completing the evaluation [28]. Whereas extensive repeated use of the app would be ideal, this is not always feasible in practice, especially when evaluating a large number of apps. Test-retest reliability showed that for most questions, results remained stable as raters presumably were more familiar with apps on the second evaluation compared with the first; however, raters were not asked how familiar they were with the app on the first evaluation. Future studies comparing AQEL ratings on first use with later evaluations after regular use of an app would be useful to corroborate this finding.

During validation, a mistake was discovered in the display logic of Q17 to Q21 in the survey. These questions all concerned the subscore of the category appropriateness to the target audience. Of the included 75 surveys 37 had been completed at the time the error was identified. The survey was corrected and updated. To account for this, the analysis of Cronbach alpha for items evaluating app appropriateness for selected age groups were taken from the second evaluation of each app.

### Comparison With Prior Work

Previous studies evaluating nutrition apps focus primarily on evidence-based features currently available in apps [11,12] and behavior change techniques or behavior theory use within apps [11,13-15]. AQEL provides the first valid and reliable instrument specifically for dietitians and nutrition researchers to evaluate the quality of apps for use in nutrition interventions. AQEL would add to such evaluations by providing a quantitative method of scoring app quality.

One app selection method in chronic disease management calls for practitioners to create an app library by identifying apps per topic. Evaluation of these apps are based on popularity and incorporation of best practice guidelines, assessing the use of behavior theory using the Behavioral Theory Content Survey [13], then matching apps to patient preferences and disease etiology [29]. This methodology, inevitably, depends heavily on the popularity rating of an app and requires an individual subjective judgment of the quality of many apps. AQEL could add to this methodology by proving an objective measure of app quality specific to content for nutrition education.

When evaluating apps in research, it has been recommended to evaluate apps in terms of what works, for which people, and in what circumstances [6]. AQEL allows for this by considering not just the app but also the end user. AQEL is consistent with a previous study evaluating platforms supporting apps, incorporating the same perspectives of developer, end user, and content provider [30].

At the onset of this study, no tools had been developed for app evaluation. During development of the AQEL tool, another app evaluation tool, the mobile app rating scale (MARS) was published for health app evaluation [28]. This 23-item tool included 5 subscales for measuring app quality: engagement, functionality, aesthetics, information, and app subjective quality. MARS also supplies optional items that can be modified to assess knowledge, attitudes, and intention to change; however, these are not included in the main scoring of MARS. Reliability testing for MARS was completed using evaluation of mental health apps with overall two-way mixed ICC=.79, 95% CI 0.75-0.83, whereas the subscales ICC=.50 to .80. Cronbach alpha of subscales=.80 to .89, median=.85, and overall scale=.90. Although not originally designed for nutrition apps, one recent study used the first four MARS categories to evaluate weight loss apps, finding that IRR between 2 raters was good, with median Krippendorf alpha=.80 and interquartile range=.14 [31].

However, AQEL differs from MARS in several important ways. First, development and reliability testing of AQEL was specifically based on input from practicing and research nutrition professionals. ICC testing in MARS relied on 2 raters’ evaluations, whereas reliability testing of the AQEL used 25 raters. PCA was used to refine AQEL, and test-retest reliability was evaluated; steps not included in the testing of MARS. Second, AQEL includes as primary constructs the categories of behavior change potential, knowledge, and skill development. These categories are not captured as part of the main MARS score; instead, there are optional items assessing similar categories: knowledge, attitudes, and intentions to change. Reliability testing of these categories is not provided for MARS. Behavior change potential, which is included in AQEL but not MARS, along with functionality and the appropriateness and quality of content for the targeted health condition, which are included in both scales, have been cited as critical for a complete evaluation of health-related apps [10]. MARS and AQEL both allow for modification of items concerning the targeted health behavior or audience; only AQEL allows for modification of items based on the targeted age group being considered. This allows greater flexibility as a dietitian could rate the same app differently when considering two different age groups. Finally, AQEL places a clear emphasis on evaluating the ability of the app to support education to increase nutrition knowledge and support behavior change.

An additional checklist was recently published for physician use in evaluating health apps; however, no information is provided on development, validation or reliability testing, and no scoring scheme was provided [32].

### Conclusions

The AQEL is a reliable tool for use when designing educational interventions that include nutrition-related apps. This tool fills a gap by allowing for standardized evaluation of the vast number of apps available for use in dietetics practice and research that have not undergone rigorous testing [6,9]. By providing evaluation based on multiple factors of quality, app selection can focus on the specific needs of the client. For example, if looking for an app specifically to support behavior change, those scores can be focused on, while also evaluating for functionality and appropriateness for the age and nutrition needs of the client. Scores from the scale can be evidence to justify app selection for interventions as well. Additionally, this tool will help inform app selection in future studies assessing for consistent use, behavior change, and improved clinical outcomes, and to provide dietitians with standardized reports [8-10] on the strengths and weaknesses of apps available to their clients.

## Appendix

Multimedia Appendix 1 Checklist for Reporting Results of Internet E-Surveys (CHERRIES).

Multimedia Appendix 2 App quality evaluation.

## Abbreviations

AQEL: app quality evaluation
ICC: intraclass correlations
IRR: interrater reliability
PCA: principal component analysis
MARS: Mobile App Rating Scale
MCAR: missing completely at random
