# Supplementary material for: A New Tool for Nutrition App Quality Evaluation (AQEL): Development, Validation, and Reliability Testing
Source: JMIR Mhealth Uhealth. 2017 Oct 27;5(10):e163. doi: 10.2196/mhealth.7441 (PMC5681720; doi:10.2196/mhealth.7441)
Supplement: Multimedia Appendix 1 [file mhealth_v5i10e163_app1.pdf]

Checklist for Reporting Results of Internet E-Surveys (CHERRIES)

Design

The App Quality Evaluation (AQEL) was designed for use by nutrition researchers and registered dietitians when evaluating of apps. For validation, three populations were targeted: nutrition professionals, app developers, and app end users. For reliability testing, nutrition educators were targeted from the Nutrition Education for the Public Dietetics Practice Group of the Academy of Nutrition and Dietetics. All samples were convenience samples.

IRB (Institutional Review Board) approval and informed consent process

IRB approval was obtained before recruiting participants. Participants were recruited through email blast, where they were informed of the study purpose, investigator information, length of time participation would require, and data storage. After participants agreed to participate, the information was reiterated in an information letter sent via email attachment along with information on how to take the survey. Written consent was waived. Emails containing personal information were deleted, and all identifying data is stored on a password protected computer or in a locked filing cabinet in the in the principal investigators laboratory.

Development and pre-testing

This paper outlines survey development in the methods section. In the second round of face validation, and in reliability testing, the survey was tested in its electronic format.

Recruitment process and description of sample having access to the questionnaire

For validation, participants were contacted by direct email or by advertising in a weekly e-blast sent to university employees. For reliability testing, members of the Nutrition Education for the Public Dietetics Practice Group of the Academy of Nutrition and Dietetics were contacted through an electronic mailing list.

This was a closed survey. For the first round of validation, participants were sent the survey as a word document. For the second round of validation and for reliability testing, only participants who responded to the recruitment email were supplied with the access link. For reliability testing a unique code was given to each participant.

Survey administration

The survey was administered using Qualtrics, with a link to access the survey supplied by email. Participation was optional. Amazon gift cards were offered as incentives for survey completions. Data was collected over 2 months with participants completing the survey for 3 apps each on 2 occasions 1 month apart.

Adaptive questioning was used, with responses to some questions determining which questions were shown later in the survey. Fifty-one items distributed over 10 screens were included in the survey, with some items grouped as a series of sub-questions. Between 1 and 12 items were on a screen. Four screens

included 1 or 2 items, five screens included 6 to 8 items (two of these could be reduced by adaptive questioning), and one screen had 12 items.

Response rates

Twenty-nine people initially agreed to complete 3 app evaluations using the tool, 25 actually completed the evaluations (86%).

Preventing multiple entries from the same individual

Each participant was required to enter a unique code. If participants started a survey but did not finish, they were required to restart the entire survey. The completed survey was kept for analysis. Participants completed the survey for three apps on two occasions for each app in the first data set, and on one occasion on in the second data set. The user codes were used to assure responses were limited to this number.

Analysis

All participants finished the survey; however some items were skipped. Multiple imputations as described in the methods were used to handle missing data.
