# Supplementary material for: A New Tool for Nutrition App Quality Evaluation (AQEL): Development, Validation, and Reliability Testing
Source: JMIR Mhealth Uhealth. 2017 Oct 27;5(10):e163. doi: 10.2196/mhealth.7441 (PMC5681720; doi:10.2196/mhealth.7441)
Supplement: Multimedia Appendix 2 [file mhealth_v5i10e163_app2.pdf]

App Quality EvaL (AQEL)

For access to the online survey, information on how to score AQEL, as well as permission to use the questions, please contact Kristen DiFilippo at kdifilip@illinois.edu.*

App Information

Q1 **The first questions are to clearly identify which app is being evaluated.**

Q2 What is the name of the app you are evaluating?

Q3 What is the version number of the app (if the app has never been updated, please put Version 1)? You will find this when downloading the app.

O Version (example: 1.2) ____

O Not sure

Q4 Is this app the full app or a lite version of the app? You will find this when downloading the app, or on the app icon.

O Full

O Lite

O Unsure/Not applicable

Q5 The most recent revision date (or date of creation if no revisions) is:

- O Within the last month
- O Within the last 6 months
- O Within the last year
- O Longer than one year
- O Date generated by application (does not apply to content)
- O Not applicable

Q6 What "store" did you use to download the app?

- O Google Play
- O iTunes
- O App Store
- O Other (please provide name of store) ____________________

End of Block

AQEL: User information

Q7 **This next set of questions is to gather user information about the app.** *Spend 10-15 minutes familiarizing yourself with and trying out all parts/features of the app. After using the app, please answer the rest of the questions. You may refer back to the app as you answer the questions.*

Q8 Please select the device(s) you used to evaluate the app (please check all that apply)

▢ iPad

▢ iPhone

▢ Kindle

▢ Android

▢ Other, please list ____

End of Block

AQEL: App Purpose

Q9 Do you feel that the app has a clear purpose?

O Yes

O Maybe

O No

Q10 Does the app title accurately describe the content of the app?

- O Title describes the content very accurately
- O Title mostly describes the content accurately
- O Title is somewhat related to the content
- O Title is hardly related to the content
- O Title does not relate to the content in any way

End of Block

AQEL: Behavior change, knowledge and skill development

Q11 In your opinion does the app try to...

	Yes	No
increase knowledge?	O	O
develop a skill?	O	O
change behavior?	O	O

Q12 Do you think the app will...

	Definitely yes	Probably yes	Maybe	Probably not	Definitely not
increase the user's knowledge?	O	O	O	O	O
lead to the development of a skill?	O	O	O	O	O
lead to behavior change?	O	O	O	O	O

Q13 When considering activities within the app, please answer the following:

	Very likely	Likely	Somewhat likely	Unlikely	Very unlikely
Will the activities help the user to increase knowledge?	O	O	O	O	O
Will the activities help the user to change behavior?	O	O	O	O	O
Will the activities help the user to develop a skill?	O	O	O	O	O

Q14 Please answer the following questions:

	Definitely yes	Probably yes	Maybe	Probably not	Definitely not
Would your friends use this app?	O	O	O	O	O
Do you intend to use this app in the future?	O	O	O	O	O
Will you do something differently after using this app?	O	O	O	O	O
Will you try to do something new after using this app?	O	O	O	O	O

Q15 How well does the app provide:

	Very well	Well	Adequately	Poorly	Very poorly
Information?	O	O	O	O	O
Feedback on progress?	O	O	O	O	O
Timely feedback whenever needed?	O	O	O	O	O

Q16 Is feedback provided when the user participates in an activity in the app?

O Yes

O No

O Not applicable

End of Block

AQEL: App functionality

Q17 Please rate the following:

	Very good	Good	Ok	Bad	Very bad
Speed of loading the app	O	O	O	O	O
The user's ability to "retrace their steps" if they need to	O	O	O	O	O
Transitions from page to page	O	O	O	O	O
Function of any animations (quick & functional - slow & fragmented)	O	O	O	O	O
Design of menus and icons	O	O	O	O	O
Ease of navigation to the app's various features	O	O	O	O	O

End of Block

AQEL: What app is for/Who app is for

Q18 **This set of questions refers to what the app is for, and who it is for.**

Q33 What age group is the app targeting? Please select all that apply

- ▢ Children
- ▢ Teenageers
- ▢ Adults
- ▢ General Audience (all of the above)
- ▢ Other (Please list) ____

Q19 Is the app appropriate for children in terms of

	Yes	Maybe	No
Maturity level	O	O	O
Nutrition needs	O	O	O
Cognitive abilities	O	O	O
Color scheme	O	O	O
Readability	O	O	O

Q37 Is the app appropriate for teenagers in terms of

	Yes	Maybe	No
Maturity level	O	O	O
Nutrition needs	O	O	O
Cognitive abilities	O	O	O
Color scheme	O	O	O
Readability	O	O	O

Q38 Is the app appropriate for adults in terms of

	Yes	Maybe	No
Maturity level	O	O	O
Nutrition needs	O	O	O
Cognitive abilities	O	O	O
Color scheme	O	O	O
Readability	O	O	O

Q39 Is the app appropriate for a general audience in terms of

	Yes	Maybe	No
Maturity level	O	O	O
Nutrition needs	O	O	O
Cognitive abilities	O	O	O
Color scheme	O	O	O
Readability	O	O	O

Q40 Is the app appropriate for the other age group you selected in terms of

	Yes	Maybe	No
Maturity level	O	O	O
Nutrition needs	O	O	O
Cognitive abilities	O	O	O
Color scheme	O	O	O
Readability	O	O	O

Q34 Who is the target audience of the app? Please select all that apply

- ☐ People seeking help for a medical condition such as diabetes, heart disease, eating disorders, or other medical conditions. (please list which condition the app is for, or list the other medical condition) ____
- ☐ People with specific nutrition concerns such as food allergies, sports nutrition, or other nutrition concerns. (please list which nutrition concern the app is for, or list the other nutrition concern) ____
- ☐ People who are shopping for food
- ☐ People seeking recipes/meal ideas
- ☐ People seeking guidance for restaurant eating
- ☐ People seeking weight loss support
- ☐ People seeking nutrition education (including nutrition education games)
- ☐ Other, (please list target audience) ____

Q20 When considering people seeking help for a medical condition

	Yes	Maybe	No
Is the app appropriate?	O	O	O
Does the app cover the topic comprehensively?	O	O	O
Does the app cover the medical condition as well as including related topics?	O	O	O
Is the level of detail provided adequate for this population's educational needs?	O	O	O

Q41 When considering people with specific nutrition concerns

	Yes	Maybe	No
Is the app appropriate?	O	O	O
Does the app cover the topic comprehensively?	O	O	O
Does the app cover the medical condition as well as including related topics?	O	O	O
Is the level of detail provided adequate for this population's educational needs?	O	O	O

Q42 When considering people who are shopping for food

	Yes	Maybe	No
Is the app appropriate?	O	O	O
Does the app cover the topic comprehensively?	O	O	O
Does the app cover the medical condition as well as including related topics?	O	O	O
Is the level of detail provided adequate for this population's educational needs?	O	O	O

Q43 When considering people seeking recipes/meal ideas

	Yes	Maybe	No
Is the app appropriate?	O	O	O
Does the app cover the topic comprehensively?	O	O	O
Does the app cover the medical condition as well as including related topics?	O	O	O
Is the level of detail provided adequate for this population's educational needs?	O	O	O

Q44 When considering people seeking guidance for restaurant eating

	Yes	Maybe	No
Is the app appropriate?	O	O	O
Does the app cover the topic comprehensively?	O	O	O
Does the app cover the medical condition as well as including related topics?	O	O	O
Is the level of detail provided adequate for this population's educational needs?	O	O	O

Q45 When considering people seeking weight loss support

	Yes	Maybe	No
Is the app appropriate?	O	O	O
Does the app cover the topic comprehensively?	O	O	O
Does the app cover the medical condition as well as including related topics?	O	O	O
Is the level of detail provided adequate for this population's educational needs?	O	O	O

Q46 When considering people seeking nutrition education

	Yes	Maybe	No
Is the app appropriate?	O	O	O
Does the app cover the topic comprehensively?	O	O	O
Does the app cover the medical condition as well as including related topics?	O	O	O
Is the level of detail provided adequate for this population's educational needs?	O	O	O

Q47 When considering the "other" target audience that you listed

	Yes	Maybe	No
Is the app appropriate?	o	o	o
Does the app cover the topic comprehensively?	o	o	o
Does the app cover the medical condition as well as including related topics?	o	o	o
Is the level of detail provided adequate for this population's educational needs?	o	o	o
